# Prognostic Value of TNF-Related Apoptosis Inducing Ligand (TRAIL) in Acute Coronary Syndrome Patients

**DOI:** 10.1371/journal.pone.0053860

**Published:** 2013-02-18

**Authors:** Pavel Osmancik, Elena Teringova, Petr Tousek, Petra Paulu, Petr Widimsky

**Affiliations:** Cardiocenter, Department of Cardiology, 3^rd^ Medical School, Charles University and University Hospital Kralovske Vinohrady, Prague, Czech Republic; Virginia Commonwealth University, United States of America

## Abstract

**Background:**

Apoptosis plays an important role in the development of heart failure. The aim of the prospectively designed study was to assess whether the concentration of apoptotic markers apoptosis-stimulating fragment (Fas, CD95/APO-1) and tumor necrosis factor-related apoptosis inducing ligand (TRAIL) can predict prognosis in patients with acute coronary syndromes.

**Methods:**

The concentrations of soluble Fas and TRAIL were determined in 295 patients with acute coronary syndromes. The status of all patients was evaluated at 6 months. The primary goal was a composite end-point of death and hospitalization for heart failure. The secondary end-points were re-MI, death alone and stroke alone.

**Results:**

During the median follow-up of 6 months, 26 patients experienced the composite end-point. Using multivariate logistic regression, the concentration of TRAIL was the strongest significant and independent predictor of composite end-point (OR 0.11 (95% CI 0.03–0.45), p = 0.002). Low concentration was associated with poor prognosis of patients. Other significant predictors of composite end-point were serum creatinine (OR 7.7 (95% CI 1.1–54.5, p = 0.041) and complete revascularization (OR 0.19 (95% CI 0.05–0.78, p = 0.02). Independent significant predictors of death in the multivariate analysis were the concentration of TRAIL (OR 0.053 (95% CI 0.004–0.744), p = 0.029), older age (OR 1.20 (95% CI 1.02–1.41, p = 0.026) and serum creatinine (OR 15.1 (95% CI 1.56–145.2), p = 0.0193). Re-MI or stroke could not be predicted by any combination of obtained parameters.

**Conclusions:**

Low concentrations of soluble TRAIL represent a strong predictor of a poor prognosis in patients with acute coronary syndrome. The predictive value of TRAIL concentration is independent of age, ejection fraction, index peak troponin level, concentration of BNP or serum creatinine.

## Introduction

Apoptosis plays an important role in the early development of heart failure and left ventricular remodeling in patients following myocardial infarction [1]. The extent of lost myocardium following acute myocardial infarction varies from patient to patient and depends on the degree of activity of apoptotic processes.

Apoptosis-stimulating fragment (Fas, CD95/APO-1) and TNF-related apoptosis-inducing ligand (TRAIL, Apo2L), both of which are members of the TNF super-family, have significantly involved in the process of apoptosis [2]. In vitro, TRAIL binds to its receptor TRAIL-R1 and TRAIL-R2, and activates caspase-8 through the Fas-associated death domain. The activated caspase-8 mediates caspase-3 activation and promotes cell death [3]. Thus, both molecules are involved in the transition of healthy into failing myocardium.

So far, several markers have been found which can predict a poor prognosis in patients with acute coronary syndrome (ACS). Among the most important and well established in patients with ACS are cardiac troponins and brain natriuretic peptide (BNP) [4]–[5]. Soluble Fas and TRAIL are also been tested in the assessment of prognostic stratification in a population of patients with chronic heart failure and in the population of elderly patients with cardiovascular disease [6]–[7]. Low concentrations of soluble TRAIL were found to be associated with poor prognoses in these particular patient groups. The aim of the present study was to assess the prognostic significance of the concentration of both molecules in patients with ACS.

## Methods

### Study population and follow-up

Study participants were prospectively enrolled in the Cardiocenter University Hospital Kralovske Vinohrady, Prague. Inclusion criterion was ACS treated using percutaneous coronary intervention (PCI). All participants were admitted due to ACS: ST-elevation myocardial infarction (STEMI), non ST-elevation myocardial infarction or unstable angina pectoris (NSTEMI/UA) with typical symptoms. Diagnoses were made based on typical symptoms, changes in electrocardiogram (ECG) and testing positive for cardiac troponins according to guidelines of the European Society of Cardiology (ESC) for the management of STEMI and NSTEMI/UA [8], [9]. All participants underwent coronary angiography with subsequent PCI; patients without revascularization could not be included in the study due to their worse prognosis compared to patients with revascularization [10]. Coronary angiography was performed immediately in patients with STEMI or in unstable patients with NSTEMI/UA, or within 48 h following admission in the remaining NSTEMI/UA patients. Exclusion criteria were the following: 1) indication for coronary artery bypass grafting (CABG) 2) no revascularization possible, and 3) life-expectancy less than 6 months due to non-cardiac reasons (malignancy, severe chronic obstructive pulmonary disease). Patients indicated for CABG were excluded due to planned surgery, which could negatively impact mortality. Echocardiography was performed in all patients on admission or on the following day. The study was approved by the local Ethics Committee and written informed content was obtained from each patient. The study protocol conforms to the ethical guidelines of the 1975 Declaration of Helsinki. Follow-up visits were arranged six months after the index procedure. Patients were seen in the outpatient department or were contacted by phone. When end-point was suspected (during the visit in the outpatient department or during a call), the patient was asked for discharge letter from the hospitalization. When a patient could not be contacted by phone, then their relatives were contacted by phone, or by a letter, and his (her) vital status was checked in health insurance service. The primary end-point was defined as a combination of death or hospitalization for heart failure within 6 months following PCI. The secondary end-points were death alone, re-MI alone or stroke alone within 6 months following PCI.

### Blood sampling for apoptotic molecules measurements and laboratory analysis

Apoptotic molecules were analyzed from venous blood samples obtained on the morning of the day following coronary angiography. Blood was drawn from the antecubital vein in 7 ml standard serum syringes. Syringes were centrifuged at 3500 rpm for 15 min; serum was stored at −70°C for batch analysis. Serum concentrations of reported molecules were measured using commercially available Enzyme-Linked ImmunoSorbent Assay (ELISA) assays (Fas and TRAIL: R&D Systems, MN, USA; BNP: USCN Life Science Inc., TX, USA). Intra-assay coefficients of variations were 2.67% for Fas, 3.36% for TRAIL and 13.2% for BNP.

The lower cut-off value was 20 pg/mL for sFas, 2.9 pg/mL for sTRAIL, and 31.2 pg/mL for BNP. The upper detection limit for BNP was 2000 pg/mL. All measurements were performed by staff who were unaware of the clinical data.

### Blood sampling for biochemistry and hematology

Blood sampling for BNP was done at the same time-point as blood sampling for Fas and TRAIL. Troponin was measured at admission, 6–12 hours later, on day 2 and in day 3. Additional sampling was done at discretion of treating physician. Blood samples for biochemistry (e.g. serum creatinine, serum urea nitrogen, glucose, liver enzymes) and hematology (e.g. hemoglobin level, leukocyte count, platelet count) were taken at admission.

### Procedural (angiographic) characteristics

Results from coronary angiographies were analyzed by two experienced cardiologists. Left main coronary stenosis was defined as angiographic evidence of ≥50% stenosis. The severity (extension) of coronary disease was defined as the number of diseased vessels (i.e. 1, 2 or 3-vessel disease). Complete revascularization was defined as the absence of any resting coronary artery stenosis exceeding 50% of diameter at time of discharge. Complete revascularization was achieved either by one PCI during the index event (for patients with single vessel disease) or by a further scheduled PCI prior to discharge. The number of stents and the total length of stents were calculated. Procedural difficulties associated with PCI angiography were defined as a combination of slow-flow phenomenon (TIMI 1 or TIMI 2 grade), no reflow (TIMI 0) or side branch occlusion. Final data processing (biochemistry, hematology, coronary angiography) was completed for 98% of the patients.

### Statistical analysis

Continuous data were tested for distribution using the Kolmogorov-Smirnov test. Continuous data with normal distribution are presented as mean ± SD, with non-Gaussian distribution as median [inter-quartile range]. Correlations between variables were assessed using the Spearman correlation coefficient. Categorical data were analyzed using χ^2^ test.

Univariate logistic regression analysis was used for studying the association between clinical, biochemical variables, medication, measured apoptotic molecules and pre-defined end-points. The clinical variables entered into the model were age, gender, history of MI, history of previous revascularization (PCI or CABG), hypertension, smoking status, and diabetes mellitus. Further variables included in the model were medication used before admission, hematological, biochemical characteristics (including troponin I and BNP values), and angiographic characteristics. Variables that showed either a significant result (p<0.05) or were near statistical significance (p<0.1) were included in the multivariate stepwise logistic regression model to determine those independently related to end-points. A receiver-operating characteristic (ROC) curve analysis was constructed to determine optimal cut-off values. Kaplan – Meier survival curves for TRAIL dichotomized at optimal ROC were constructed, and statistical signifikance was calculated using log rank test. All tests were 2-sided, and a p value <0.05 was considered statistically significant. All data analyses were performed using SPSS version 15.0 software (SPSS Inc., Chicago, Illinois).

## Results

### Clinical results

Three-hundred and twenty patients were enrolled in the study. Follow-up was completed for 295 patients, complete information was not available for remaining 25 patients. While information from the health insurance service confirmed the patients were not dead, we had no way of assessing the other end-point of the study. Therefore their data was not analyzed. Twenty-six (8.8%, group End-point) of 295 patients met at least one of pre-defined end-points within 6 months of follow-up. Twelve (4.1%) had died, and 14 (4.7%) had been hospitalized for heart failure. Additionally, eleven (3.7%) patients had undergone re-MI and 3 (1%) had had a stroke. The remaining 269 patients did not experience any of the pre – specified endpoints (group End-point free). Baseline clinical characteristics, index event characteristics, medications at admission, important laboratory parameters and angiographic characteristics of both groups (i.e. End-point and End-point free) are summarized in Table 1.

**Table 1:** Characteristics of studied patients regarding their medical history, index event, medication on admission, and basic laboratory parameterst.

	Combined end-point (n = 26)	End-point free (n = 269)	p value
Age (yrs.)	72.6±10.8	66.1±13.4	<0.05
Male gender	20 (76.9)	192 (71.4)	n.s.
BMI	27.8±4.4	29.1±20.6	n.s.
DM	9 (34.6)	71 (26.4)	n.s.
AF	3 (11.5)	31 (11.5)	n.s.
Hypertension	17 (65.4)	149 (55.4)	n.s.
Smoking status	15 (57.7)	159 (59.1)	n.s.
History of MI	9 (34.6)	58 (21.6)	n.s.
Beta blocker	8 (30.7)	100 (37.2)	n.s.
ACEI	11 (42.3)	117 (43.5)	n.s.
Aspirin	11 (42.3)	95 (35.3)	n.s.
Statin	8 (30.8)	83 (30.9)	n.s.
STEMI	12 (46.2%)	145 (53.9)	n.s.
Killip class	1.87±1.2	1.13±0.5	<0.001
LV EF	40.5±12.2	48.9±11.3	<0.001
Hemoglobin (g/dl)	130.9±22.6	138.6±24.9	n.s.
Leukocyte count (*10^9^/l)	16.6±27.4	10.4±3.7	<0.001
Thrombocytes (*10^12^/l)	228.6±79.1	224.6±57.6	n.s.
Serum creatinine (µmol/l)	160.5±148.8	87.5±28.1	<0.001
Glucose (mmol/l)	9.1±4.1	7.6±3.5	n.s.
ALT (µkatl/l)	0.95±1.1	0.96±1.9	n.s.
Left main disease	5 (19)	15 (6)	<0.05
CAD severity	2.19+0.94	1.91±0.81	0.09
Complete revascularization	6 (23)	149 (55)	0.002
Number of stents	1.73±1.31	1.30±0.58	0.002
Length of stents	30.19+ 26.19	22.45±11.43	0.005
Procedural difficulties	1(4)	12 (4)	n.s.

BMI – body mass index, DM – the presence of diabetes mellitus, AF – the presence of atrial fibrillation during index hospitalization, smoking status – smoking before admission, STEMI – myocardial infarction with ST-segment elevation, LV EF – ejection fraction of left ventricle, glucose – the concentration of glucose at admission, ACEI – the admission of angiotensin – converting enzyme blockers at discharge, aspirin – the admission of aspirin at discharge, statin – the admission of statin at discharge, ALT – alanine aminotransferase, CAD severity – the extension of coronary artery disease, Complete revascularization – the absence of any stenosis of 60% or more in at least one coronary artery at discharge.

### The concentration of apoptotic molecules

**Figure 1:**
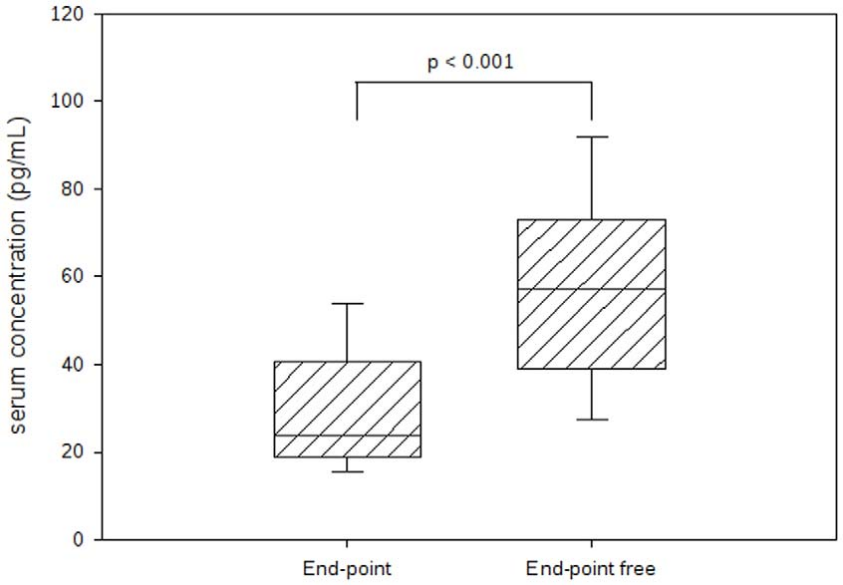
Serum concentration of soluble TRAIL. Data are expressed as median with interquartile ranges. Statistical comparison was done by Wilcoxon test.

The concentration of Fas was higher in the End-point group (7440 [5774–9443] pg/mL vs. 6530 [5702–8009] pg/mL) in the End-point free group; however, this difference was not statistically significant. The concentration of sTRAIL was significantly lower in the End-point group (23.7 [19.2–40.4] pg/mL vs. 57.1 [38.9–72.9] pg/mL in the End-point free group, p<0.001, Figure 1). End-point patients also had higher concentrations of BNP: 1699 [1238–2200] pg/mL vs. 297 [60–977] pg/mL, p<0.001), higher peak troponin I levels: 148.2±146.8 ng/mL vs. 59.6±77.2 ng/mL, p<0.001, serum creatinine: 160.5±145.8 µmol/L vs. 87.5±28.1 µmol/L, p<0.001), and leukocyte count: 16.6±27.3 vs.10.4±3.7, p<0.001.

### The correlation between markers of apoptosis and necrosis

There was an inverse correlation between peak troponin I levels and the concentration of sTRAIL (r = −0.335, p<0.001). The concentration of sTRAIL correlated inversely with the concentration of leukocyte count (r = −0.220, p<0.001), and positively with LV EF (r = 0.315, p<0.001). There was no correlation between the level of BNP with sFas (r = 0.0728, p = 0.29) or sTRAIL (r = −0.126, p = 0.066).

### Primary endpoint: death and heart failure

In the univariate regression model, the following variables were significantly (or almost significantly, p<0.01 at least) associated with the combined end-point death or hospitalization for heart failure: age, Killip class, a need for mechanical ventilation, ejection fraction of left ventricle (LV EF), peak troponin level, BNP, serum creatinine, serum urea nitrogen, leukocyte count, hemoglobin level, serum glucose, the concentration of Fas and the concentration of TRAIL, severity of coronary artery disease (i.e. number of diseased vessels), left main disease, complete revascularization, number of stents and total length of stents. Exact numbers are shown in Table 2. All these parameters were next tested in a stepwise multiple logistic regression model. In the multivariate analysis, most important significant predictor of the combined end-point was the concentration of TRAIL (OR 0.11 (95% CI 0.03–0.45), p = 0.002). Low concentration was associated with poor prognosis of patients. Other significant predictors of combined end-point were serum creatinine (OR 7.7 (95% CI 1.1–54.5, p = 0.041), complete revascularization (OR 0.19 (95% CI 0.05–0.78, p = 0.02), and on borderline level, the concentration of BNP (OR 1.56 (95% CI 0.96–2.53, p = 0.07).

**Table 2:** Univariate analysis of predictors of combined end-point (death or hospitalization for heart failure).

	odds ratio	95% confidence interval	p
TRAIL	0.07	0.025–0.193	<0.001
Fas	6.77	1.39–32.78	0.018
BNP	1.88	1.25–2.83	0.002
Troponin peak	1.17	0.98–1.39	0.078
Killip class	3.03	1.94–4.71	<0.001
AF at admission	1.20	0.39–3.74	0.748
STEMI	0.73	0.32–1.64	0.451
Mechanical ventilation	6.86	1.54–30.54	0.011
Age	1.06	1.02–1.10	0.008
Male gender	1.31	0.51–3.41	0.567
BMI	0.99	0.91–1.09	0.978
DM	1.60	0.68–3.75	0.283
Hemoglobin	0.96	0.94–0.98	0.003
Serum creatinine	24.0	6.82–84.66	<0.001
Urea nitrogen	1.93	1.04–3.61	0.038
Glucose	2.66	0.96–7.36	0.059
ALT	0.88	0.44–1.76	0.721
AST	1.18	0.78–1.78	0.437
Leukocytes	2.33	1.06–5.82	0.069
LV EF	0.94	0.91–0.98	<0.001
Left main disease	4.03	1.33–12.18	0.013
CAD severity	1.53	0.93–2.53	0.096
Complete revascularization	0.24	0.09–0.62	0.003
Number of stents	1.90	1.20–3.01	0.006
Length of stents	1.03	1.01–1.06	0.008
Procedural difficulties	1.02	0.75–1.37	0.910

The table shows selected characteristics, which were included in the univariate regression analysis. All variables, that approached statistical significance (p<0.1) were included in the multivariate stepwise logistic regression model.

Troponin peak – peak troponin level during hospitalization, AF – the presence of atrial fibrillation at admission or anytime during index hospitalization, STEMI – myocardial infarction with ST-segment elevation, BMI – body mass index, Glucose – glucose at admission, ALT – alanine aminotransferase, AST – aspartate amino transferase, LV EF – left ventricular ejection fraction, CAD severity – the extension of coronary artery disease, Complete revascularization – the absence of any stenosis of 50% or more in at least one coronary artery at discharge, Procedural difficulties – the combination of slow flow, no reflow od side branch occlusion during PCI.

### Secondary endpoint: death

In the univariate regression model, the following variables were significantly (or almost significantly) associated with the occurrence of death and were entered into the multiple logistic model: age, the presence of diabetes, Killip class on admission, LV EF, BNP level, leukocyte count, hemoglobin level, serum creatinine, glucose on admission, complete revascularization, and the concentration of TRAIL and Fas (exact numbers are shown in Table 3). All these parameters were next tested in a stepwise multiple logistic regression model. In the multivariate analysis, significant predictors of death were the concentration of TRAIL (OR 0.053 (95% CI 0.004–0.744), p = 0.029), older age (OR 1.20 (95% CI 1.02–1.41, p = 0.026) and serum creatinine (OR 15.1 (95% CI 1.56–145.2), p = 0.0193).

**Table 3:** Univariate analysis of predictors of death.

	odds ratio	95% confidence interval	P
TRAIL	0.07	0.014–0.31	0.001
Fas	8.21	0.67–100.2	0.056
BNP	2.24	0.98–5.13	0.056
Age	1.13	1.05–1.21	0.001
Killip class	3.67	2.20–6.13	<0.001
Male gender	1.17	0.31–4.42	0.820
BMI	0.95	0.83–1.09	0.461
DM	3.04	0.95–9.74	0.061
Smoking status	0.48	0.15–1.56	0.222
Hypertension	1.09	0.34–3.52	0.883
Serum creatinine	14.92	3.63–61.34	<0.001
Leukocytes	3.97	1.26–12.49	0.019
Hemoglobin	0.96	0.93–0.98	0.007
LV EF	0.96	0.91–1.00	0.067
AF	1.19	0.25–5.67	0.829
Troponin peak	1.13	0.89–1.43	0.322
Glucose	4.81	1.22–19.05	0.025
Complete revascularization	0.17	0.037–0.789	0.024

Characteristics included in the univariate regression analysis are shown. All variables, that approached statistical significance (p<0.1) were included in the multivariate stepwise logistic regression model.

BMI – body mass index, DM – diabetes mellitus, Smoking history – actual smoking status at admission, Hypertension – history of hypertension, LV EF – left ventricular ejection fraction, AF – the presence of atrial fibrillation at admission or anytime during index hospitalization, Troponin peak – peak troponin level during hospitalization, Glucose – glucose at admission, Complete revascularization – the absence of any stenosis of 50% or more in at least one coronary artery at discharge.

### Secondary endpoint: re-MI

Re-MI occurred in 11 patients within 6 months of follow-up. In the univariate regression model, only the concentration of TRAIL and maximum troponin level were significantly associated with re-MI and were therefore entered into the multiple logistic model. However, in a stepwise multiple logistic regression model, none from above mentioned parameters was significant predictor of re-MI.

### Secondary endpoint: stroke

Only 3 (1%) patients underwent a stroke during follow-up of six months. Therefore, this endpoint could not been sufficiently statistically analyzed.

### Receiver operating characteristic analysis

**Figure 2:**
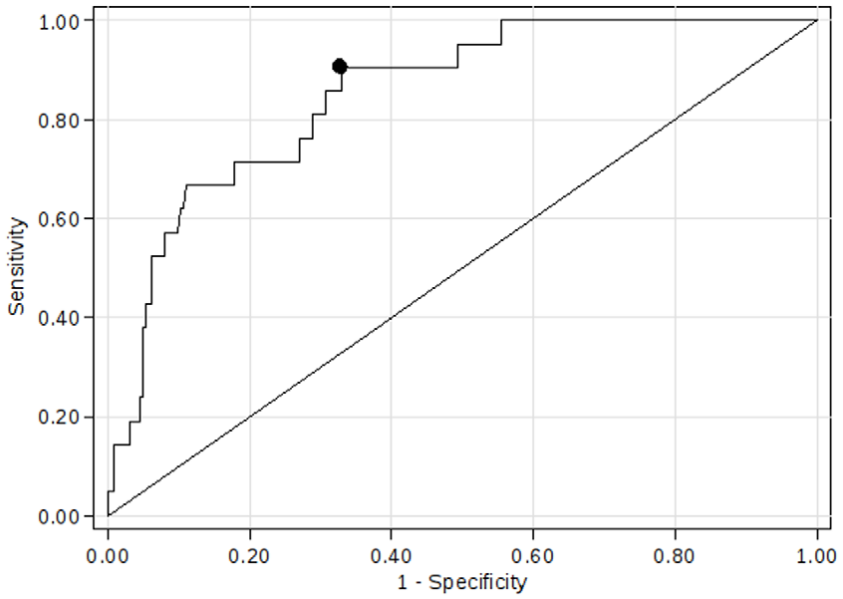
Receiver-operating characteristic curve for the concentration of soluble TRAIL in relation to the primary end-point (death and heart failure). The closed black dot on the curve shows the concentration of TRAIL (44.6 ng/mL) with the optimal combination of sensitivity and specificity.

**Figure 3:**
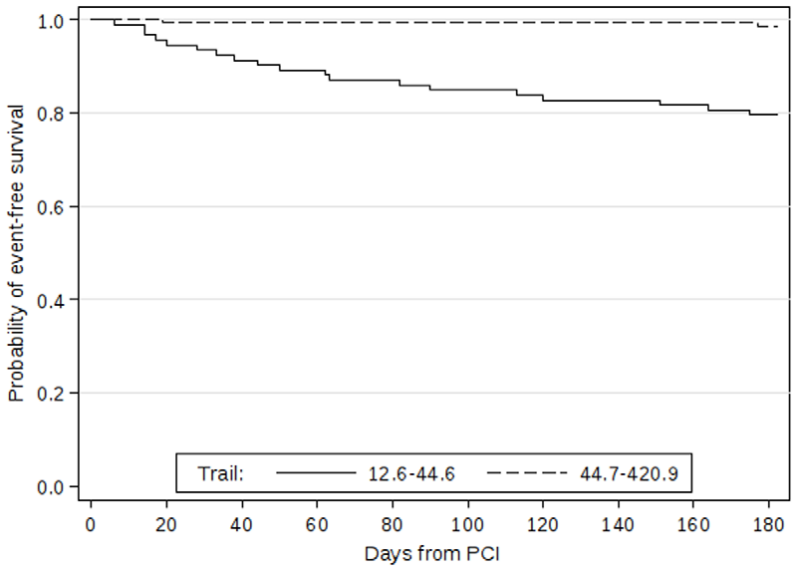
Kaplan – Meier survival curves event rate in patients grouped according to calculated optimal cut-off value of TRAIL. Patients with TRAIL concentrations up to 44.6 ng/mL are shown as a solid curve, patients with TRAIL concentrations higher than 44.6 ng/mL are shown as a dotted curve. P<0.001 (log rank test).

Receiver operating characteristic curve analysis demonstrated that the concentration of soluble TRAIL was able to distinguish between patients with and without subsequent combined end-point (area under the curve 0.85, 95% CI 0.78–0,93, p<0.001; Figure 2). A concentration of TRAIL of 44.6 ng/mL was identified as the optimal cut-off to predict the combination of death and heart failure within 6 month follow-up, providing a sensitivity of 90.5 (95% CI 69.6–98.8), a specificity of 67.1% (95% CI 60.6–73.2), a negative predictive value of 98.7% (95% CI 95.4–99.8), and a positive predictive value of 20.4% (95% CI 12.8–30.1%). A Kaplan – Meier survival curves of patients relative to the calculated optimal concentration of TRAIL are shown in Figure 3. The differences between survival curves was statistically significant (p<0.001, log rank test).

## Discussion

Heart failure resulting from ACS is one of the leading causes of death in western countries [11]. Invasive strategies including emergent or early angiography and PCI in patients with ACS is associated with a decreased rate of death, heart failure or re-MI [10]. Despite revascularization, the morbidity and mortality of patients with ACS remains high. In our study, the combined endpoint of re-MI, death or stroke was reached 8.8% of studied patients. Several prognostic markers have been found to predict high-risk patients and improve their prognosis. Among them, cardiac troponins and BNP have been established in several studies and are routinely used in the clinical practice [4]–[5].

Apoptosis plays an important role in left ventricular remodeling. The extent of apoptosis differs from patient to patient and is associated with the level of left ventricular remodeling following myocardial infarction. Abbate et al. showed that the degree of left ventricular remodeling was directly associated with the extent of apoptosis in subjects who died shorty (10 days) after STEMI [1]. Moreover, the *ex vivo* measured apoptotic activity in human sera is higher in patients shorty after an MI and can predict survival in patients with heart failure [12]. Apoptotic cardiomyocyte loss after infarction is also known to be variable, and its severity and extent can be modulated by several pathophysiological mechanisms and variables, such as the renin-angiotensin-aldosterone system or adrenergic stimulation [13]–[14]. In an experimental animal model, the administration of anti-apoptotic protein (hepatocyte growth factor) was associated with decreased apoptosis and left ventricular remodeling [15]. Clinical studies seem to suggest that the pharmacological benefits of ACE inhibition and β-adrenergic receptor blockade, in human heart failure, are at least partly attributable to interruption of apoptosis [16]–[17].

The exact molecular mechanism of function and role of TRAIL in the cardiovascular system is not completely understood. Studies using *in vitro* tumor cell lines have shown that TRAIL binding to TRAIL-receptor 1 or –2 leads to initiation of the caspase cascade that leads to apoptotic cell death [18]–[19]. The effect of TRAIL on non-tumor cells is not clear. Some authors found that soluble TRAIL can selectively kill the tumor cells without apparent toxicity to normal cells in mouse animal models [20]. However, others have described that soluble TRAIL, *in vitro*, also induces apoptosis and proinflammatory activity in normal human umbilical vein endothelial cells (HUVEC) [21]. While others have shown that TRAIL, *in vitro*, on HUVEC exerts anti-inflammatory activity (by increasing nitric oxide and prostanoid production) [22]. TRAIL might play an important role in modulating leukocyte/endothelial cell adhesion. *In vitro* exposure of HUVEC to TRAIL has been associated with a significant reduction in the pro-adhesive activity of endothelial cells relative to neutrophils in response to inflammatory cytokines [23].

The effect of TRAIL can be mediated by reducing pro-inflammatory activity, which is present during ACS and is associated with a worse prognosis. Moreover, in animal models, direct administration of recombinant TRAIL reduced the development of cardiomyopathy in a diabetic mouse model [24].

In humans, recent cross-sectional and prospective studies suggest an inverse association between serum TRAIL levels with the severity of coronary artery disease and with adverse outcomes in patients with heart failure. In older patients with prevalent cardiovascular disease, low levels of TRAIL were associated with increased risk of death over a period of 6 years [7]. Niessner et al. measured serum Fas and TRAIL in 360 patients with advanced chronic heart failure (NYHA III or IV) who had been admitted to hospital due to heart failure decompensation, and followed them for 16 months [6]. In a mulivariate analysis, higher concentrations of Fas were associated with higher risk for combined end-point of death and heart failure, but not for death alone. Although TRAIL concentration were not able to predict the occurrence of the combined end-point in the multivariate model, TRAIL was a very strong inverse predictor of death. In our study, Fas was a predictor of the composite end-point in univariate analysis, but lost its significance in the multivariate mode. TRAIL was an independent predictor of both death and the composite end-point. Compared to our study, the study by Niessner et al. was done with a different patient population, which included patients with chronic heart failure irrespective of etiology (45% were ischemic). Although the number of patients in our study was lower, our patient population was much more homogenous (100% ischemic etiology). This can explain the small differences in results between our study and Niessner's study. Michowitz et al. showed that serum levels of soluble TRAIL, but not Fas, were reduced significantly in patients with ACS compared to patients with stable atherosclerotic disease and healthy subjects [25]. Thus, TRAIL might be more specific for patients with ischemic etiology of left ventricular dysfunction relative to other etiologies. Secchierro et al. found significantly lower concentrations of serumTRAIL in patients after MI (measured within 24 hours after MI, which was similar to the time-point of measurement in our study) compared to healthy subjects [26]. Moreover, low concentrations of TRAIL were associated with higher incidences of death or heart failure at the 1-year follow-up. The number of patients enrolled in the study by Secchiero et al. was small (only 60 patients with MI), which means that especially data regarding prediction must be viewed cautiously. The predictive power of our results, based on a substantially larger population, is significant in that it confirms that low concentrations of TRAIL, in patients following an ACS, is a strong marker of death and heart failure. As it can be seen in Kaplan – Meier curve, the distribution of incidence of end-point was similar during the entire follow- up. Another recent paper by Secchiero et al. demonstrated that a high ratio between serum osteoprotegerin and TRAIL, in patients with acute MI, was associated with higher risk of developing heart failure [27]. The exact mechanism of the negative impact of higher TRAIL concentration on the prognosis of patients following MI is not known. However, there is agreement, based on recent trials, regarding the positive impact of low concentrations of TRAIL on patients prognoses.

Precise measurement of cardiac apoptosis can only be done with cardiac tissue samples. Although scientifically interesting, it cannot be done routinely in clinical practice. Therefore, the search for (serum) biomarkers of apoptosis that are indicative of actual tissue level apoptosis as well as being indicative of clinical prognoses, is of great importance. Several markers of apoptosis have been found, that can be measured from peripheral blood, such as Fas, TRAIL, and tumor necrosis factor – α. However, the question regarding which provides the greatest predictive power and which would be the best for use in clinical practice or interventional studies remains unanswered. The only way forward is through assessments done using large observational studies. Our study was a step toward answering the question. If confirmed, prospective interventional studies would be needed to determine if TRAIL-guided treatment can improve the prognosis of patients following MI.

Experimental data have shown that programmed cell death after myocardial injury contributes in a major way to ventricular remodelling and the development of heart failure. Rapid reperfusion by PCI or thrombolysis helps to minimize acute ischemic injury. However, apoptosis seems to have stronger association with reperfusion than hypoxia [28]. Fortunately, there appears to be a therapeutic window for interrupting excessive apoptosis, which can be days or weeks after the acute ischemic insult [29]. The finding of reliable apoptosis biomarkers or methods to positively affect the process of left ventricular remodeling after MI (e.g. by new antiapoptotic drugs) could serve to improve patient prognoses. Despite the ambiguity of TRAIL at the molecular level, in clinical practice, lower concentrations have been found to be associated with a poor prognosis in several recently published trials.

Several reports have indicated that serum creatinine is also a negative prognostic indicator for MI patients [30]. Our findings are in agreement with them. Cardiac troponins are known prognostic factors associated with poor prognoses in patients with ACS [4]. Higher concentrations of troponin I or T are associated with higher mortality in patients with STEMI and NSTEMI [31], [32]. Our findings are in complete agreement with these findings. Importantly, the concentration of the apoptotic molecule TRAIL correlated inversely with the concentration of troponin and positively with the LV EF in our patients. Thus, even though LV remodeling, after an MI, can take weeks or months, pathologically low concentrations seem to be present from the first day following MI. Therefore, low concentrations of TRAIL could present a reduced inhibition power against apoptosis. Moreover, low concentrations of TRAIL remained a predictor of poor outomes, independent of troponin concetrations.

### Study Limitation

One limitation of our study was related to sample size. Additionally, 9% of patients were lost during follow-up, which could have influenced our results. The completion of presented data was 98%. The concentrations of apoptotic molecules were measured only once; additional samples would have been useful and could have provided a better understanding the role of these molecules in the development of heart failure.
